# Rapid localization of ureteral calculi in patients with renal colic by “ultrasonic ureteral crossing sign”

**DOI:** 10.1038/s41598-020-58805-x

**Published:** 2020-02-05

**Authors:** Jianguo Xia, Junhong Peng, Gang Wang, Tao Zheng, Qi Xu

**Affiliations:** Wuhan Fourth Hospital, Puai Hospital, Tongji Medical College, Huazhong University of Science and Technology, Wuhan, China

**Keywords:** Urinary tract obstruction, Ureter

## Abstract

In this study, the term “ultrasonic ureteral crossing sign” is defined, and the diagnostic accuracy of this sign in the rapid localization of ureteral calculi is assessed. Between January 2017 and June 2018, 535 patients underwent ultrasound examination for suspected ureteral calculi. The “ultrasonic ureteral crossing sign” was classified as either positive or negative and correlated with the location of ureteral calculi. Of the 451 patients who were ultimately diagnosed with ureteral calculi, 263 patients had a positive sign, of which 258 patients had distal ureteral calculi, and 188 patients had a negative sign, of which 164 patients had proximal ureteral calculi. Eighteen stones were located in the ureter across the iliac vessels. For patients with a positive “ultrasonic ureteral crossing sign”, we observed a 91% sensitivity, 97% specificity, 98% PPV, 87% NPV, and AUC of 0.94 for distal ureteral calculi. For patients with a negative “ultrasonic ureteral crossing sign”, we observed a 97% sensitivity, 91% specificity, 87% PPV, 98% NPV, and AUC of 0.94 for proximal ureteral calculi. The “ultrasonic ureteral crossing sign” was found to accurately predict the location of ureteral calculi, significantly improve the efficiency of ultrasound examination, and provide a useful basis for follow-up treatment.

## Introduction

Acute renal colic caused by ureteral calculi is one of the most common abdominal emergencies, accounting for 1% of emergency department visits^1^. Non-contrast computed tomography (NCCT) is the main examination method and gold standard. However, repeated CT scans can lead to excessive radiation exposure^2^. In recent years, with the focus on reducing radiation exposure, more attention has been paid to the diagnostic value of ultrasound (US) for ureteral calculi. According to the European Association of Urology (EAU) 2019 Guidelines, “Ultrasound should be used as the primary diagnostic imaging tool”^3^.

US diagnosis of ureteral calculi is less sensitive than NCCT^4^ and provides limited clinical information when ureteral calculi are not found directly. Although some indirect findings (e.g., hydronephrosis, hydroureter, perirenal effusion, etc.) suggest the possibility of ureteral calculi, NCCT is often required to determine the location and size of the calculi. New US signs need to be identified to provide more useful information to guide follow-up diagnosis and treatment and to further reduce the use of NCCT.

**Figure 1:**
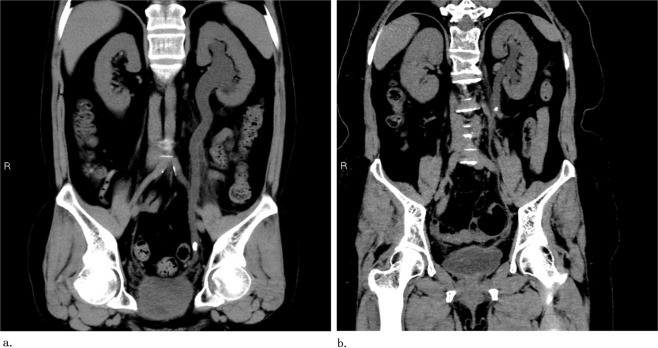
(**a**) NCCT showing distal ureteral calculi leading to ureteral dilatation across the iliac vessels. (**b**) NCCT showing that proximal ureteral calculi do not cause ureteral dilatation across the iliac vessels.

The ureter is divided into proximal and distal segments, with the ureter crossing the iliac vessels as the boundary^5^. When the stone moves into the ureter, the ureter proximal to the stone dilates due to the obstruction, while the ureter distal to the stone is not affected (Fig. 1). Therefore, the dilatation of the ureter across the iliac vessels can indicate that the obstruction is located in the distal ureter.

A normal ureter is invisible on US, but a dilated ureter appears as an anechoic tubular structure, which can be easily displayed against the background of the iliac vessels. We consider the phenomenon of a dilated ureter spanning from the front of the iliac vessels in US examination as a positive “ultrasonic ureteral crossing sign”. Although this phenomenon has likely been observed by many US specialists in their daily work, its value for revealing the location of the ureteral calculi has not been reported or analyzed.

The purpose of this study was to assess the diagnostic accuracy of the “ultrasonic ureteral crossing sign” in distinguishing proximal and distal ureteral calculi.

## Results

A total of 451 cases of ureteral calculi were ultimately included in the study. The mean age of the patients was 43.2 years (range, 17–95 years), 97 (21.5%) were women (mean age, 46.6 years; age range, 18–81 years), and 354 (78.5%) were men (mean age, 42.3 years; age range, 17–95 years), in accordance with the epidemiological characteristics of ureteral calculi. Of the 451 ureteral calculi, 262 (58.1%) were located in the left ureter, 189 (41.9%) were located in the right ureter, 344 ureteral calculi were confirmed by US, and 107 ureteral calculi were confirmed by NCCT.

Of the 245 patients with a positive “ultrasonic ureteral crossing sign”, 240 were located in the distal ureter (181 cases were confirmed by US, 59 cases by NCCT), and 5 were located in the proximal ureter (all confirmed by NCCT). Of the 188 patients with a negative “ultrasonic ureteral crossing sign”, 164 cases were located in the proximal ureter (138 cases were confirmed by US, 26 cases by NCCT), 24 cases were located in the distal ureter (7 cases were confirmed by US and 17 cases by NCCT), and 18 cases were located in the ureter across the iliac vessels and diagnosed as mid-ureteral calculi.

A positive “ultrasonic ureteral crossing sign” diagnosis of distal ureteral calculi had a sensitivity of 91% (95% confidence interval [CI]: 86.8%, 94.1%) and a specificity of 97% (95% CI: 93.2%, 99%). The ROC AUC value was 0.94 (0.91 to 0.96). The positive predictive value was 98% (95% CI: 95.3%, 99.1%), and the negative predictive value was 87% (95% CI: 82.3%, 90.9%) (Table 1). The negative “ultrasonic ureteral crossing sign” diagnosis of proximal ureteral calculi had a sensitivity of 97% (95% CI: 93.2%, 99%) and a specificity of 91% (95% CI: 86.8%, 94.1%). The ROC AUC value was 0.94 (0.91 to 0.96). The positive predictive value was 87% (95% CI: 82.3%, 90.9%), and the negative predictive value was 98% (95% CI: 95.3%, 99.1%) (Table 2).

**Table 1:** Test characteristics of positive “ultrasonic ureteral crossing sign” in predicting distal ureteral calculi.

	Distal ureteral calculi	Proximal ureteral calculi
Ultrasonic ureteral crossing sign (+)	240	5
Ultrasonic ureteral crossing sign (−)	24	164

CI, confidence interval; LR, likelihood ratio.

Sensitivity = 91% (95% CI [86.8, 94.1]); Specificity = 97% (95% CI [93.2, 99]); AUC = 0.94(0.91–0.96).

Positive Predictive Value = 98% (95% CI [95.3, 99.1]); Negative Predictive Value = 87% (95% CI: [82.3, 90.9]).

**Table 2:** Test characteristics of a negative “ultrasonic ureteral crossing sign” in proximal ureteral calculi.

	Proximal ureteral calculi	Distal ureteral calculi
Ultrasonic ureteral crossing sign (−)	164	24
Ultrasonic ureteral crossing sign (+)	5	240

CI, confidence interval; LR, likelihood ratio.

Sensitivity = 97% (95% CI [93.2, 99]); Specificity = 91% (95% CI [86.8, 94.1]); AUC = 0.94(0.91–0.96).

Positive Predictive Value = 87% (95% CI [82.3, 90.9]); Negative Predictive Value = 98% (95% CI: [95.3, 99.1]).

In addition, we performed statistical analysis on patients whose ureteral stones were not directly found by ultrasound, and finally confirmed by NCCT. In these patients, a positive “ultrasonic ureteral crossing sign” diagnosis of distal ureteral calculi had a sensitivity of 78% (95% confidence interval [CI]: 66.6%, 86.4%) and a specificity of 84% (95% CI: 96.3%, 94.5%). The ROC AUC value was 0.81 (0.72 to 0.88). The positive predictive value was 92% (95% CI: 84%, 96.4%), and the negative predictive value was 60% (95% CI: 49.5%, 70.5%). The negative “ultrasonic ureteral crossing sign” diagnosis of proximal ureteral calculi had a sensitivity of 84% (95% CI: 96.3%, 94.5%) and a specificity of 78% (95% confidence interval [CI]: 66.6%, 86.4%). The ROC AUC value was 0.94 (0.91 to 0.96). The positive predictive value was 60% (95% CI: 49.5%, 70.5%), and the negative predictive value was 92% (95% CI: 84%, 96.4%).

## Discussion

The position of the iliac vessels is relatively fixed and is a good positioning marker for US examination. Because it appears as an anechoic tubular structure on US imagery, it provides contrast with any strong echoes in front of it (such as a stone or dilated ureter), making it quite easy to observe the presence of a ureter or stone crossed in front of the iliac vessels. According to our experience, typical US radiologists can master the judgment of an “ultrasonic ureteral crossing sign” in a short period of time.

When the stone moves into the ureter, the ureter proximal to the stone dilates due to the obstruction. Therefore, the positive “ultrasonic ureteral crossing sign” indicates that the stone is located in the distal ureter. Our results show that a positive “ultrasonic ureteral crossing sign” has a high sensitivity (91%), extremely high specificity (97%) and positive predictive value (98%) for distal ureteral calculi. A negative “ultrasonic ureteral crossing sign” suggested that the stone was located in the proximal ureter, but its specificity was lower than that of a positive “ultrasonic ureteral crossing sign,” which was 91% in this study. There are two possible reasons for this: small stones (especially those located at the end of the ureter) are sometimes insufficient to obstruct/dilate the ureter, and due to improper manipulation by the sonologist or the poor abdominal condition of the patient (such as excessive obesity or excessive gas in the intestine), the dilated ureter could not be identified.

Adverse conditions such as obesity and intestinal gas will not only affect the display of ureteral calculi, but also affect the display of “ultrasonic ureteral crossing sign”, thereby making the originally positive “ultrasonic ureteral crossing sign” appear false negative. In order to analyze the impact of these adverse conditions on the “ultrasonic ureteral crossing sign”, we made a statistical analysis in the cases that the stones could not be directly displayed by ultrasound and were located by CT: in these cases, the sensitivity (78%) and specificity (84%) of the positive “ultrasonic ureteral crossing sign” to the distal ureteral calculi were decreased, but they still remained a high positive predictive value (92%); the negative predictive value of “ultrasonic ureteral crossing sign” for proximal ureteral calculi were reduced to 60%. This indicates that the adverse abdominal conditions will make the negative “ultrasonic ureteral crossing sign” to locate the proximal ureteral calculi less valuable, but the positive “ultrasonic ureteral crossing sign” can still locate the distal ureteral calculi well.

The main problem with the use of US for the diagnosis of ureteral calculi is the dependence on the experience and techniques of the radiologist. To locate ureteral calculi on imaging, sonologist usually need to examine the ureter carefully from beginning to the end, a process that often takes a long time and requires constant hand pressure to reduce intestinal gas interference^6^. Thus, radiologists need to devote more energy during the exam, leading to a greater risk of work-related musculoskeletal disorders (WMSDs)^7^. In addition, patients may experience additional suffering. Therefore, it is of great significance to find a sensitive and specific method to locate ureteral calculi, improve the efficiency of US examination, shorten the examination time, reduce the workload of radiologists, and reduce patient suffering. In view of its high positive predictive value, for patients with a positive “ultrasonic ureteral crossing sign”, we can focus most of the examination on the distal ureter, while the proximal ureter needs only a simple and rapid scan to exclude multiple stones. A total of 258 patients with a positive “ultrasonic ureteral crossing sign” were enrolled in this study. According to an average time savings of 4.5 minutes per patient, a total of 1161 minutes of examination time could have been saved. For patients with a negative “ultrasonic ureteral crossing sign”, it may not significantly shorten the examination time, but it allows us to focus more on the proximal ureter, thus indirectly improving the effectiveness of US examination.

With the focus on reducing radiation exposure, various guidelines currently recommend US as the primary diagnostic imaging tool for ureteral calculi to reduce the use of CT. But when US failed to find ureteral calculi, clinicians had little information other than the possible presence of ureteral calculi. Clinicians make follow-up treatment methods based on the location and size of the stones, these patients may still have to undergo NCCT. The “ultrasonic ureteral crossing sign” can further indicate whether the stone is located in the proximal or distal ureter. This information is useful for clinicians because proximal/distal stones may lead to different follow-up treatment methods, thereby further reducing the use of NCCT.

Stones located in the distal ureter are important predictive factors of spontaneous passage^8^. Without considering the size of the stones, distal ureteral calculi have a higher rate of spontaneous passage (perhaps because a stone that is difficult to spontaneously pass also has difficulty reaching the distal ureter). At the same time, some studies have shown that some drugs and appropriate sexual behavior can effectively promote the spontaneous passage of distal ureteral calculi^9,10^. Therefore, for patients with a positive “ultrasonic ureteral crossing sign”, even if US examination does not detect the stone directly, the patient may be instructed to try conservative treatment and observation without further NCCT examination. In the interventional treatment of distal ureteral calculi, most guidelines recommend ureteroscopy as a first-line treatment, regardless of the size of the stone^5^. If the patient wishes to receive or has other complications that require interventional treatment, a CT examination at the appropriate time before treatment could be chosen by urologists rather than immediate emergency CT.

The management for proximal ureteral calculi is closely related to the stone size: smaller stones have a higher rate of spontaneous passage, while larger stones are often difficult to pass spontaneously and need interventional treatment, and the treatment algorithm is also related to the size of the stones^3^. Therefore, the size of proximal ureteral calculi has very important guiding significance for follow-up treatment. For patients with a negative “ultrasonic ureteral crossing sign”, we should try our best to find and measure the stone to guide follow-up treatment, and the distal ureter should be probed if necessary. If stones cannot be found by US examination, an emergency NCCT scan may be needed. In patients with proximal ureteral calculi during conservative treatment, if a “ultrasonic ureteral crossing sign” is found to be positive in the reexamination, it indicates that the position of the stone is moved downward, which can enhance the confidence of conservative treatment.

Although not the focus of this study, the sensitivity of US in the diagnosis of ureteral calculi in our study was 76.3% (344 of 451), which was higher than the average of 45% reported in similar literature. This may be due to the following reasons: First, unlike many other countries, it is the sonologist, not the sonographer, who performs US examinations in China. Meanwhile, in an attempt to include underserved populations, lowering the cost burden has been one of our primary concerns. Our sonologists must exert extra effort to locate and measure ureteric calculi and, consequently, have sharpened their skills. Second, some patients who were excluded from the study group after negative US might have had stones, leading to an overestimation of sensitivity. Finally, in the design of the study, we assumed that the specificity of US diagnosis of ureteral calculi was 100%, but in actuality, there was a possibility of misdiagnosis. Thus, the misdiagnosis of ureteral calculi by US in our study is possible, leading to an overestimation of sensitivity.

There were some limitations to our study that should be noted. First, our study is a retrospective study and as such may be subject to confounding or bias. Second, although it has high specificity (94%) for ureteral calculi^3^, US is not a recognized benchmark diagnostic tool as NCCT. Ideally, all patients would have undergone both US and CT to ensure the most accurate sensitivity and specificity. Third, some patients with equivocal US findings refused a follow-up NCCT, which led to incomplete data. Fourth, a full bladder may lead to ureter dilatation, resulting in a false positive “ultrasonic ureteral crossing sign”. Therefore, the conclusions of this study may not be applicable to patients with a full bladder. Finally, there is the theoretical possibility of concurrent proximal and distal ureteral calculi. No such case was found in this study, so we could not assess whether such calculi will form the “ultrasonic ureteral crossing sign”. For the sake of safety, it is necessary for patients who choose conservative treatment without CT examination to receive regular US examinations until the stone has passed.

In conclusion, the “ultrasonic ureteral crossing sign” can quickly and accurately predict the location of ureteral calculi, significantly improve the efficiency of US examination, and provide additional evidence for the choice of treatment options, which has extremely important clinical significance.

## Materials and Methods

This study was a retrospective study and was approved by the Regional Ethics Committee of Puai Hospital. Due to its retrospective nature, informed consent was waived. All methods were performed in accordance with the relevant guidelines and regulations.

### Study sample

Our institution is a tertiary general hospital. Most patients with renal colic are treated in the emergency department. All patients with lumbar or flank pain suspected of acute renal colic underwent an ultrasound-CT examination in our emergency department. Patients who are suspected of having renal colic are given priority for US examination, and NCCT is recommended for patients with equivocal US findings. Our sample included all patients who underwent US examinations for suspected ureteral calculi from January 1, 2017, to June 30, 2018.

**Figure 2:**
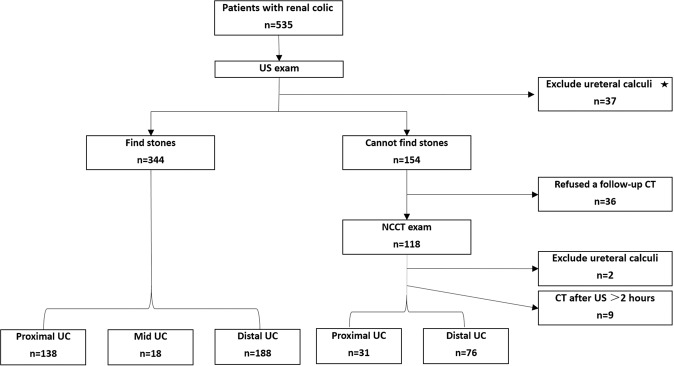
Flowchart of the patient characteristics and algorithm used (UC, ureteral calculi); ⋆: A diagnosis excluding calculi was made by the diagnosis of another cause that would justify the pain (e.g., acute appendicitis, gynecological pathology, biliary colic, etc.).

A total of 535 patients underwent US examinations to assess ureteral calculi during the study period. Of the 535 patients, 84 (15.7%) had ureteral calculi excluded by either US or NCCT or by having equivocal US results but no subsequent CT examination within 2 hours. Therefore, 451 of the 535 patients (84.3%) were diagnosed with ureteral calculi by US or NCCT and thus met the inclusion criteria. Figure 2 presents the patient characteristics.

The PACS system was searched to confirm that examinations had been performed because of the clinical suspicion of ureteral calculi. Imaging reports were reviewed and classified as positive or negative for the “ultrasonic ureteral crossing sign” and compared with the location of ureteral calculi.

### Imaging algorithm

Renal colic is managed as an urgent condition at our institution. Our imaging algorithm is implemented 24 hours a day. All patients with lumbar or flank pain suspected of acute renal colic follow the same imaging algorithm.

A staged imaging algorithm has been implemented for years in our emergency department. With this algorithm, US and NCCT are performed in patients suspected of having ureteral calculi, with US as the initial modality followed by NCCT when US findings are equivocal. For this study, US findings were considered equivocal if ureteral calculi were not identified and if either (a) there were secondary US signs of ureteral calculi (hydronephrosis, dilation of the ureter origin, or perirenal effusion) or (b) there was high clinical concern for ureteral calculi. A diagnosis excluding calculi was made by the diagnosis of another cause that would justify the pain (e.g., acute appendicitis, gynecological pathology, biliary colic, etc.). NCCT is recommended for all patients with equivocal US findings. The patient retained the right to choose whether they underwent NCCT.

### US technique

US was performed with either a Mindray Resona 7, a Mindray DC 8, or a Philips IU22 US imaging system. A convex transducer was used to assess the urinary system. All US examinations of the urinary system were performed by a registered diagnostic medical sonologist.

US examination of the kidneys focuses on whether there are kidney stones and hydronephrosis and is not the purpose of this study, nor is it described in detail.

In January 2017, the members of our practice made a commitment to observe and record whether a “ureteral crossing sign” was present before the formal US examination in patients suspected of having ureteral calculi.

**Figure 3:**
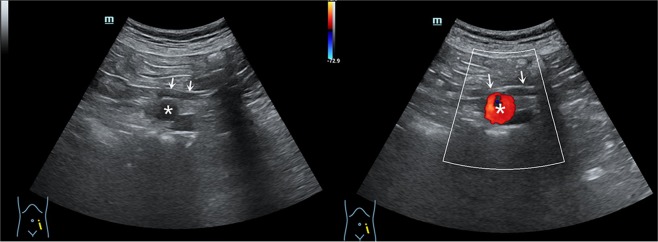
US showing a “ureteral crossing sign” as the presence of a dilated ureter (arrow) crossing the iliac vessels (*).

We defined a positive “ultrasonic ureteral crossing sign” as the presence of a dilated ureter crossing the iliac vessels in the groin region. US imaging was characterized as follows (Fig. 3):

- The anechoic tubular structure spanned from the front of the iliac vessels;
- The diameter of the tube was greater than 3 mm; and
- Color Doppler showed that there was no blood flow.

When the ureter was examined, it was probed proximally to distally. The patients were mainly placed in the supine position. The left lateral position, the right lateral position and the prone position were supplemented as needed. It should be noted that it is necessary to properly pressurize the intestine to reduce gas interference during the examination. A definitive diagnosis of calculus was given based on the following:

- Clear visualization of the calculus on sonogram; and
- Twinkling artifact on Doppler imaging.

The duration of the examination was determined on a case-by-case basis. Our sonologist typically required 3–6 minutes to examine the proximal ureter and 3–5 minutes to examine the distal ureter.

Although a filled bladder can improve the detection rate of the distal ureteral calculi, patients with acute renal colic often have urinary tract irritation. Filling the bladder takes a long time and causes great suffering to the patients. Therefore, we do not require a filled bladder.

### NCCT technique

NCCT examinations were performed with a 128-slice CT system (Philips Ingenuity Core CT scan, Philips, location Netherlands). Patients underwent NCCT craniocaudally with the following parameters: 120 kVp, automatic milliampere modulation (maximum 250 mA), slice thickness: 5 mm without interval. Axial images were reconstructed into 1.25 mm slice thickness. Coronal and sagittal images were reconstructed. No oral or intravenous contrast material was administered.

### Image interpretation

US images were stored and evaluated by the radiology resident who performed the US examination. Another radiologist examined the image through our institutional PACS system.

All NCCT images were independently reviewed by two radiologists with 20 years and 5 years of experience in radiology, respectively, who were blinded to the clinical information of the patients.

Cases of interobserver disagreement were resolved by consensus.

### Statistical analysis

Statistical analysis was performed with the Statistical Package for the Social Sciences (SPSS) version 20 for Windows. The sample size was calculated as follows:

Because the sensitivity and specificity of the “ultrasonic ureteral crossing sign” are more than 80%, we used the following formula:

$${\rm{n}}={\left[\frac{57.3\times {Z}_{1-\frac{{\rm{\alpha }}}{2}}}{\arcsin ({\rm{\delta }}/\sqrt{{\rm{p}}(1-{\rm{p}})}}\right]}^{2}$$

where Z = 1.96, α = 0.05, σ = 0.05, and p = 0.9; n = 136.89.

The sensitivity, specificity, positive predictive value, negative predictive value and AUC of the “ultrasonic ureteral crossing sign” were examined using statistical diagnostic testing.

The datasets generated during and/or analyzed during the current study are available from the corresponding author on reasonable request.
